# Quantification method of ctDNA using cell-free DNA methylation profile for noninvasive screening and monitoring of colon cancer

**DOI:** 10.1186/s13148-024-01708-9

**Published:** 2024-07-19

**Authors:** Hyojung Ryu, Ji-Hoon Kim, Yeo Jin Kim, Hahyeon Jeon, Byoung-Chul Kim, Yeonsu Jeon, Yeonkyung Kim, Hyebin Bak, Younghui Kang, Changjae Kim, Hyojin Um, Ji-Hye Ahn, Hwi Hyun, Byung Chul Kim, Inho Song, Sungwon Jeon, Jong Bhak, Eon Chul Han

**Affiliations:** 1 Clinomics, Inc., Ulsan, 44919 Republic of Korea; 2 GenomeLab, Korean Genomics Center (KOGIC), Ulsan National Institute of Science and Technology (UNIST), Ulsan, 44919 Republic of Korea; 3 Department of Biomedical Engineering, College of Information and Biotechnology, Ulsan National Institute of Science and Technology (UNIST), Ulsan, 44919 Republic of Korea; 4 Division of Colorectal Surgery, Department of Surgery, Dongnam Institute of Radiological and Medical Sciences, Busan, 46033 Republic of Korea; 5 Geromics Inc., Suwon, 16229 Republic of Korea; 6 Personal Genomics Institute (PGI), Genome Research Foundation (GRF), Cheongju, 28160 Republic of Korea

**Keywords:** Colon cancer, ctDNA, Epigenetic diagnosis, Liquid biopsy, Postoperative monitoring

## Abstract

**Background:**

Colon cancer ranks as the second most lethal form of cancer globally. In recent years, there has been active investigation into using the methylation profile of circulating tumor DNA (ctDNA), derived from blood, as a promising indicator for diagnosing and monitoring colon cancer.

**Results:**

We propose a liquid biopsy-based epigenetic method developed by utilizing 49 patients and 260 healthy controls methylation profile data to screen and monitor colon cancer. Our method initially identified 901 colon cancer-specific hypermethylated (CaSH) regions in the tissues of the 49 cancer patients. We then used these CaSH regions to accurately quantify the amount of circulating tumor DNA (ctDNA) in the blood samples of these same patients, utilizing cell-free DNA methylation profiles. Notably, the methylation profiles of ctDNA in the blood exhibited high sensitivity (82%) and specificity (93%) in distinguishing patients with colon cancer from the control group, with an area under the curve of 0.903. Furthermore, we confirm that our method for ctDNA quantification is effective for monitoring cancer patients and can serve as a valuable tool for postoperative prognosis.

**Conclusions:**

This study demonstrated a successful application of the quantification of ctDNA among cfDNA using the original cancer tissue-derived CaSH region for screening and monitoring colon cancer.

**Supplementary Information:**

The online version contains supplementary material available at 10.1186/s13148-024-01708-9.

## Introduction

Colon cancer is the third most common cancer worldwide (10.0%) and the second most common cause of death (9.4%) among cancers [1]. The annual incidence and mortality rates are continuously increasing, and by 2040, it is predicted that there will be 3.2 million new cases and 1.6 million deaths in 185 countries [1, 2]. This emphasizes the importance of early detection and monitoring of colon cancer. The most standardized method for diagnosing colorectal cancer is colonoscopy, which is invasive and involves a complex procedure and time-consuming process with low patient compliance [3]. Fecal immunochemical test (FIT) and fecal occult blood test (FOBT) are noninvasive methods based on stool samples. However, these approaches undermine their advantages by reducing the accuracy of prediction due to the effects of other intestinal diseases [4, 5]. CEA (carcinoembryonic antigen) and CA19-9 are noninvasive serological markers used for colon cancer surveillance; however, they are constrained by their limited sensitivity [6–8]. A recent study reported that among participants who declined the invasive option of colonoscopy, 97% opted for noninvasive screening, with 83% of this group expressing a preference for blood-based tests [9]. Consequently, it is necessary to develop more effective technologies for noninvasive blood-based colon cancer diagnosis and monitoring with higher accuracy, especially for predicting the tissue of origin.

Recently, circulating tumor DNA (ctDNA) in blood, which carries genetic or epigenetic alterations originating from tumors, has been actively investigated for its clinical applications as noninvasive diagnostic biomarkers for cancer [10–14]. Furthermore, since the ctDNA profile is blood-based, it can dramatically reduce the burden on patients compared to imaging and invasive biopsy. Also, the ctDNA profile can be employed for dynamically monitoring molecular changes in tumors related to therapy and after surgery [15, 16]. In general, for cancer patients, ctDNA typically exists in a relatively low proportion, ranging from 0.01% to 1.0% of the total cfDNA [17]. Various strategies exist for detecting circulating tumor DNA (ctDNA) in the blood of cancer patients, with DNA mutation or methylation-based approaches being particularly notable. The method relying on somatic mutations encounters challenges in effectively detecting ctDNA due to tumor heterogeneity and the limited presence of DNA fragments originating from tumors that contain the target mutations in plasma samples [18]. In contrast, profiling the cancer-specific aberrant methylation pattern distributed across the entire genome offers heightened clinical sensitivity and the capability for multiple detections. The detection of ctDNA based on the comprehensive methylation profile of the entire genome holds promise for effective early diagnosis, recurrence monitoring and other aspects of cancer management [19, 20]. The approach of quantifying ctDNA based on DNA methylation for cancer detection has been substantiated for its efficacy in previous studies [21–24]. Mary L. Stackpole *et. al.* have suggested that the hypermethylation profile associated with cancer is highly valuable for cancer detection, while the hypomethylation profile is suggested to be useful for detecting the tissue of origin (TOO) [10]. There was another study that introduced the concept of methylation haplotype blocks (MHB), considering the methylation status of adjacent CpG sites for the systematic discovery of markers [25]. However, this method has not been applied to the quantification of tumor-derived DNA fragments.

In a previous study in 2022, we developed the ctDNA candidate count index (ctCandi) which measures the amount of ctDNA in blood, based on the methylation density of cfDNA [26]. Using this method, we presented models that can distinguish lung cancer patients and healthy controls with mean area under the curve (AUC) of 0.925. In the present study, we applied ctCandi with a new quantification method of scoring using cancer-specific hypermethylated (CaSH) region for screening and monitoring colon cancer. We utilized tissues from 49 colon cancer patients to define the CaSH region. To evaluate the clinical application of ctDNA quantification based on the CaSH region, we employed 160 plasma samples, including pre-operation and post-operation follow-up observations from colon cancer patients, as well as 260 plasma samples from healthy controls. CaSH region-based ctDNA detection and scoring method first defines colon cancer-specific hypermethylated regions from the patient tissues and measures the relative amount ctDNA in the blood of the same patient. Machine learning models were constructed for distinguishing colon cancer patients from the control by comparing the normalized ctDNA count of CaSH regions. Furthermore, we suggested the utility of CaSH region-based ctCandi in postoperative patient prognosis monitoring.

## Results

### Quantification of ctDNA using genome-wide colon cancer-specific hypermethylated (CaSH) regions

**Fig. 1:**
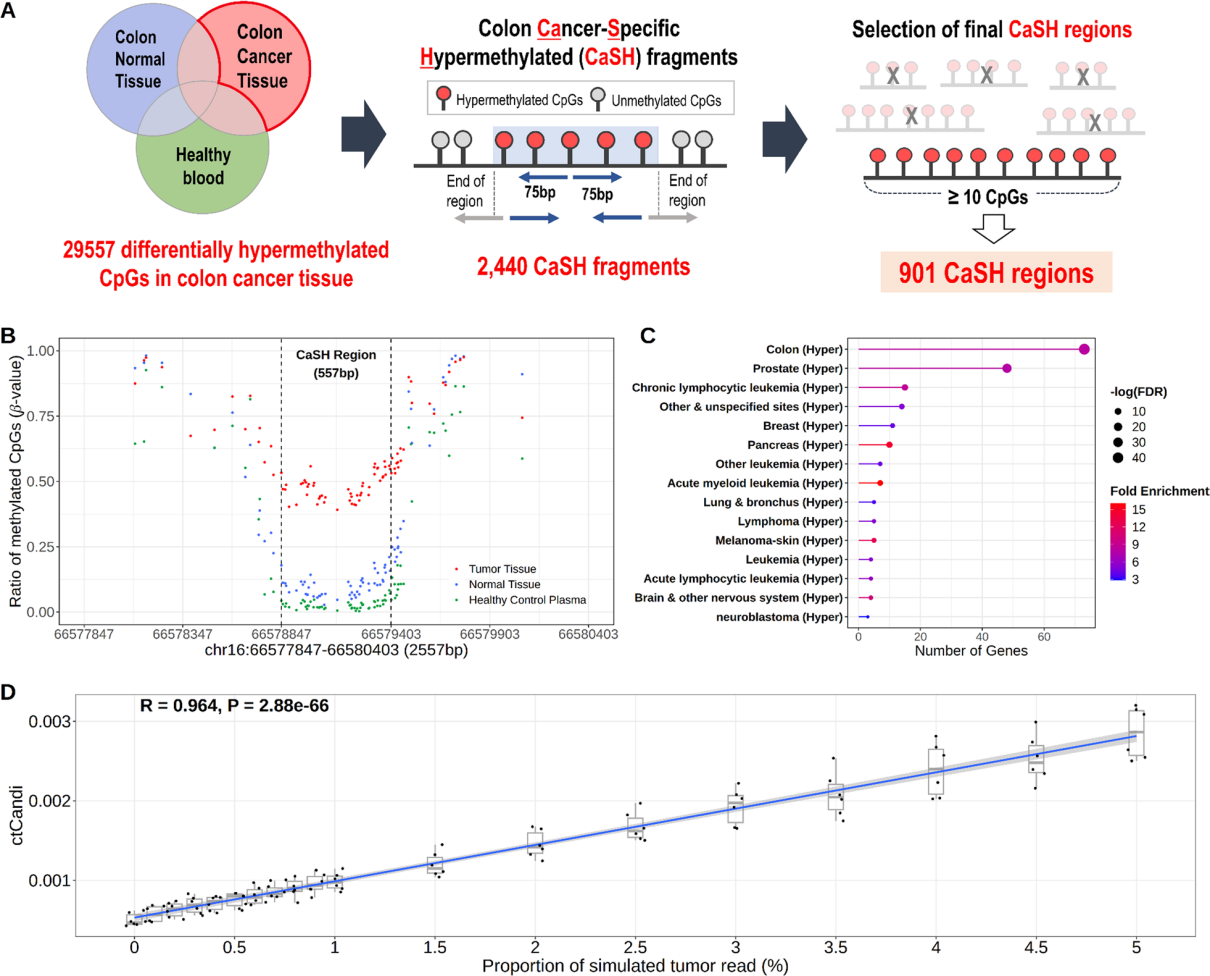
Identification of colon cancer-specific hypermethylated (CaSH) regions. **A** Schematic diagram of the definition of the CaSH regions. **B** Examples of the CaSH region extended 1 kb upstream/downstream on chr16. Dashed lines indicate start and end position of the CaSH region. **C** Gene set enrichment analysis of the CaSH regions. **D** An in silico simulation of quantification of ctDNA method, ctCandi

We defined 901 colon CaSH regions through genome-wide methylation analysis with 49 colon cancer patients and 190 healthy controls to quantify ctDNA of colon cancer (Fig. 1). The defined regions were utilized for quantifying ctDNA through the calculation of ctCandi in the blood. CtCandi estimates the amount of cancer-derived DNA fragments using the methylation profiles, as we introduced in the previous study on lung cancer [26]. To define the 901 regions, two stages of analysis were performed. Firstly, we identified 29,557 differentially hypermethylated CpGs (*β*_tumor tissue_–*β*_normal tissue_ > 0.3 and *β*_healthy plasma_ < 0.05, FDR < 0.05) by comparing colon tumor tissues to normal tissues and healthy control plasma. In previous studies, Δ*β* was primarily set at 0.2 as a threshold [10, 12]; however, we used a threshold of 0.3 to select more reliable colon cancer-specific hypermethylated CpG sites. Then, we combined adjacent hypermethylated CpG sites to generate variable length CaSH fragments using the 29,557 CpGs by taking 75 bp up- and downstream stretches (Fig. 1A) generating 2440 fragments. We further filtered out 1539 fragments that have fewer than ten hypermethylated CpG sites. Finally, we selected the last 901 CaSH regions for application to quantify colon cancer-derived ctDNA and downstream analyses. The adjoined CaSH regions range in length from a minimum of 52 bp to a maximum of 1966 bp, with an average length of 397 bp. As shown in Figs. 1B and S1A, we present examples of CaSH regions where CpG sites showed significantly higher *β* values in colon cancer tissue compared to both normal tissue and healthy plasma. The *β* value differences were diminished on the outside of the defined CaSH regions. The 901 CaSH regions showed a high proportion within CpG islands (80.6%) (Fig. S1B). Moreover, these regions were predominantly located in the exons, introns and promoters of 797 genes. When compared to the CpG distribution in the human genome, they exhibited a high proportion in these regions (Fig. S1C). Also, the 797 genes contained the highest number of genes (*N* = 73) related to colon cancer compared to other cancer types in MethCancerDB with the most significance in the enrichment test (FDR = 2.85 × 10^–43^, fold enrichment = 8.188; Fig. 1C). The 797 genes were functionally enriched with transcription, such as specifically linked to sequence-specific DNA binding (GO:0043565) and the activity of DNA-binding transcription factors (GO:0003700) in gene ontology (GO) enrichment analysis (Fig. S1D).

To validate 901 CaSH regions in distinguishing colon cancer, we analyzed Infinium Methylation 450 K array data of 263 colon cancer tissue samples and 35 colon normal tissue samples from The Cancer Genome Atlas (TCGA) and 656 healthy blood samples from Gene Expression Omnibus (GEO) dataset. Among the CpG sites on the array, 1676 CpGs from 730 CaSH regions overlapped with the 901 CaSH regions. The mean *β* value of the 1676 CpGs within the CaSH region was highest in colon cancer tissue, showing a significant difference compared to the methylation profiles of both colon normal tissue and healthy blood (Wilcoxon rank-sum test; colon normal tissue: *P* = 1.5 × 10^–21^, healthy blood: *P* = 6.9 × 10^–124^; Fig. S2A). In contrast, we found that the mean *β* value of the 439,311 CpGs outside the CaSH regions on the array was hypermethylated in healthy blood compared to both colon cancer tissue and colon normal tissue (Wilcoxon rank-sum test; colon cancer tissue: *P* = 2.5 × 10^–85^, colon normal tissue: *P* = 6.0 × 10^–23^; Fig. S2B). Additionally, we investigated the distribution of *β* values from the array data in the longest CaSH region (1966 bp) (Fig. S2C). Six CpGs were located in this region, and the *β* value profiles observed in the TCGA and GEO datasets consisted of the distribution found in both tissue and plasma from our dataset. To evaluate the consistency between the ctCandi, based on the 901 CaSH regions, and proportion of cfDNA fragments originating from colon tumors, we performed an in silico test using six simulated cfDNA data. This evaluation employed plasma samples from ten healthy controls and tumor tissue from six colon cancer patients. ctCandi was highly correlated statistically with the simulated cancer DNA ratio (Spearman’s correlation; *ρ* = 0.964, *P* = 2.88 × 10^–66^; Fig. 1D).

**Fig. 2:**
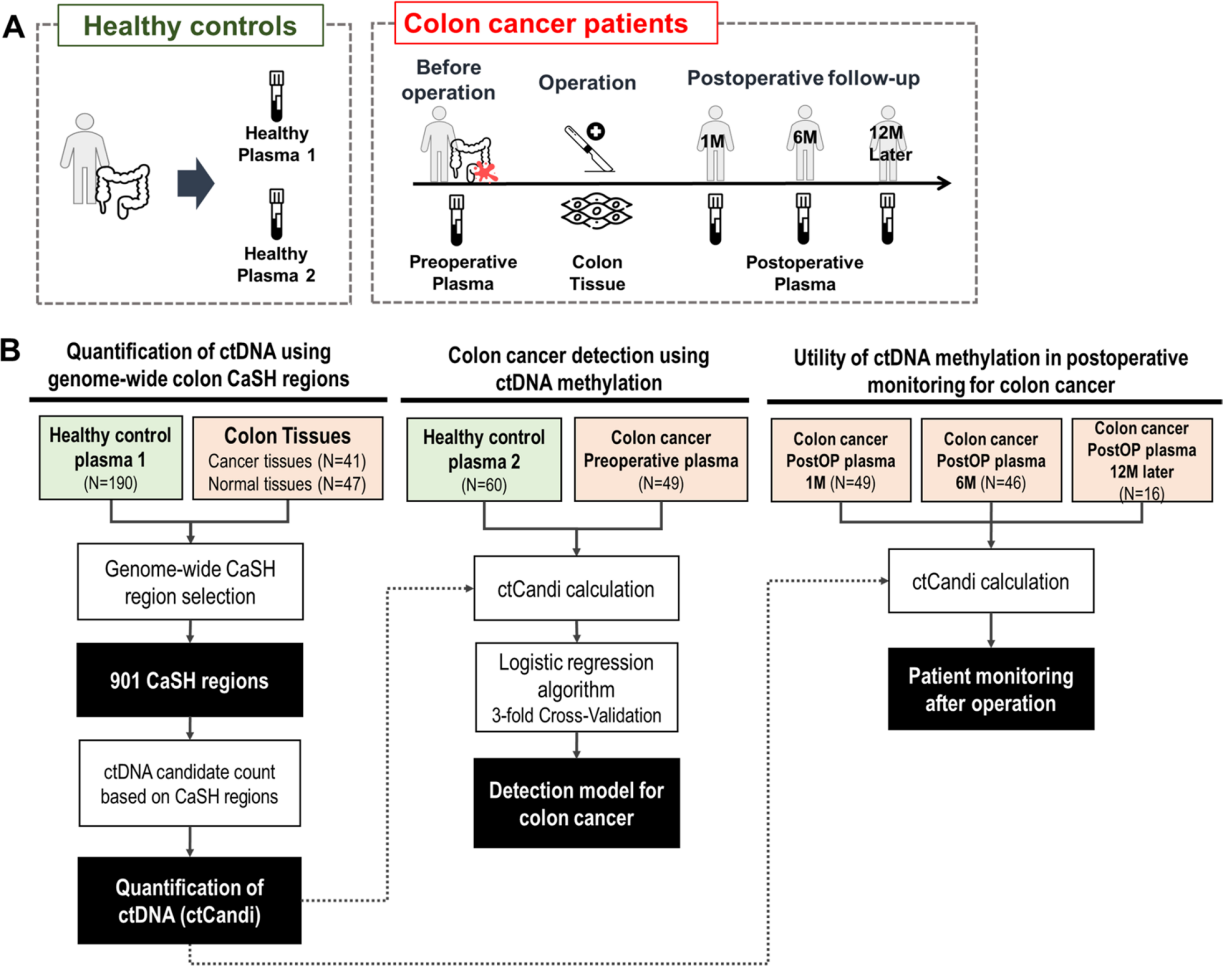
Overall study design. **A** Composition of clinical samples in healthy controls and colon cancer patients. **B** Comprehensive process flowchart for definition of CaSH regions, detection model for colon cancer and postoperative monitoring; ‘PostOP’ indicates ‘Post-operation’; ‘ctCandi’ indicates ‘ctDNA candidate count index’

The quantification method for colon cancer-derived ctDNA has been applied to the screening and monitoring of colon cancer (Fig. 2). We constructed a cancer classification machine learning model using ctCandi for the selected regions as an input feature. Independent plasma samples from colon cancer patients (*N* = 49) and healthy controls (*N* = 60) were used, and threefold cross-validation was performed. Moreover, the plasma samples collected after the patient's operation (*N* = 111 in total) were employed to assess the effectiveness of ctDNA methylation in monitoring the colon cancer patients (Fig. S3). As a healthy control group, 260 samples of plasma cfDNA were selected from the Korean Genome Project (KGP) [27]. Detailed clinical characteristics for all the collected samples are summarized in Table 1.

**Table 1:** Baseline characteristics

	Colon cancer	Healthy controls
	*n* = 49	*n* = 260
Sample type	Plasma, tissue	Plasma
Age, avg. (min, max)	65.5 (42, 86)	62.6 (40,86)
Male, *n*(%)	26 (53.1)	138 (53.1)
Female, *n*(%)	23 (46.9)	122 (46.9)
BMI(kg/m^2)	23.1	24.9
*Smoking status*		
Never	28 (57.1)	125 (59.5)
Former	16 (32.7)	59 (28.1)
Current	5 (10.2)	26 (12.4)
NA, *n*	0	50
Recurrence, *n*(%)	6 (12.2)	
*Stage, n(%)*		
II	18 (36.7)	
III	31 (63.3)	
*Chemotherapy, n(%)*		
Yes	38 (77.6)	
No	11 (22.4)	
Median F/U period (month)*	20	
Median time to recurrence (month)**	13.5	

*All patients

**Only recurrence cases

### Colon cancer detection using ctDNA methylation

**Fig. 3:**
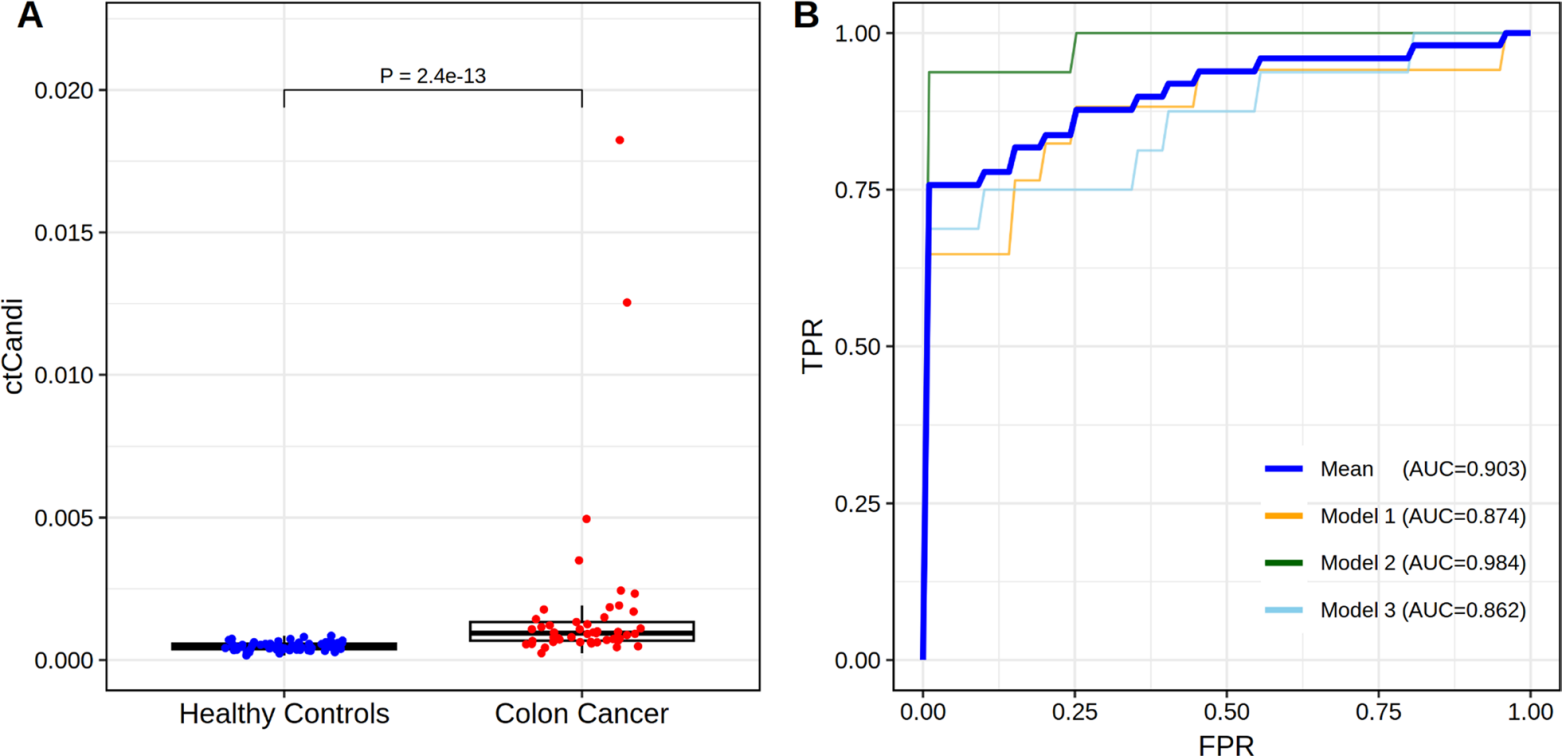
Performance of ctDNA candidate count index (ctCandi) and classification machine learning models. **A** ctCandi of 49 colon cancer patients and 60 healthy controls. The *P-*value was calculated by the Wilcoxon rank-sum test. **B** Receiver operating characteristic (ROC) curves of ctCandi for distinguishing the colon cancer patients and the healthy controls. TPR indicates a true positive ratio, and FPR indicates a false positive ratio

The logistic regression-based machine learning model for colon cancer detection demonstrated outstanding performance with an AUC of 0.903 by utilizing normalized ctDNA count of the 901 CaSH regions. Notably, the ctCandi values employed in constructing the model were significantly higher in the 49 colon cancer patients compared to the 60 healthy controls with a *P*-value of 2.4 × 10^–13^ (Wilcoxon rank-sum test; Fig. 3A). Contrasting with the previously presented single CpG-based ctCandi calculation approach, the CaSH region-based method in this study revealed a more pronounced difference between colon cancer patients and healthy controls (Wilcoxon rank-sum test, *P* = 1.3 × 10^–11^, Fig. S4). Additionally, stage III patients showed a more significant difference with the healthy controls compared to stage II patients (Wilcoxon rank-sum test; stage II: *P* = 9.0 × 10^–7^, stage III: *P* = 5.4 × 10^–10^; Fig. S5). Furthermore, we classified 49 patients into microsatellite instability (MSI) and microsatellite stable (MSS) groups, categorizing them based on the presence or absence of *RAS*, *KRAS* and *NRAS* gene mutations to calculate CaSH-based ctCandi (Fig. S6). By confirming a significant difference (Wilcoxon rank-sum test; *P* = 1.1 × 10^-17^) between the MSS group and healthy controls (Fig. S6A), we distinguished colon cancer patients from healthy controls, independent of MSI status. Although the MSI group showed a significant difference compared to both the MSS and healthy control groups (Wilcoxon rank-sum test; MSS: *P* = 0.0045, healthy control: *P* = 0.0039), the analysis was limited by the large difference in sample size (MSI = 3, MSS = 46). Among the three genes, only *NRAS* showed a significant difference between wild-type and mutation groups (Wilcoxon rank-sum test; *P* = 0.027; Fig. S6B). However, in this comparison as well, the statistical power of the analysis was constrained by a substantial difference in sample sizes between the two groups (wild type = 46 and mutation = 3).

The models were validated using a threefold cross-validation approach during the model training step. All these models performed well in distinguishing colon cancer patients from healthy controls with an average AUC of 0.903 (0.862–0.984), an average sensitivity of 82% (74–95%) and an average specificity of 93% (80–100%) (Fig. 3B). Furthermore, a significantly positive correlation emerged between tumor size and ctCandi, indicating an association with increased ctDNA quantity attributed to a higher tumor burden (*ρ* = 0.53, *P* = 8.2 × 10^–5^; Fig. S7).

The differentially hypomethylated regions in colon cancer were notably fewer (*N* = 48) than the hypermethylated regions (*N* = 901). The limited number of regions failed to substantially distinguish between colon cancer patients and healthy controls (Wilcoxon rank-sum test, *P* = 0.4, Fig. S8A). The logistic regression models that employed the hypomethylated regions as input features had low discriminative power with 0.495 of a mean AUC (Fig. S8B).

### The utility of ctDNA methylation in postoperative monitoring for colon cancer

**Fig. 4:**
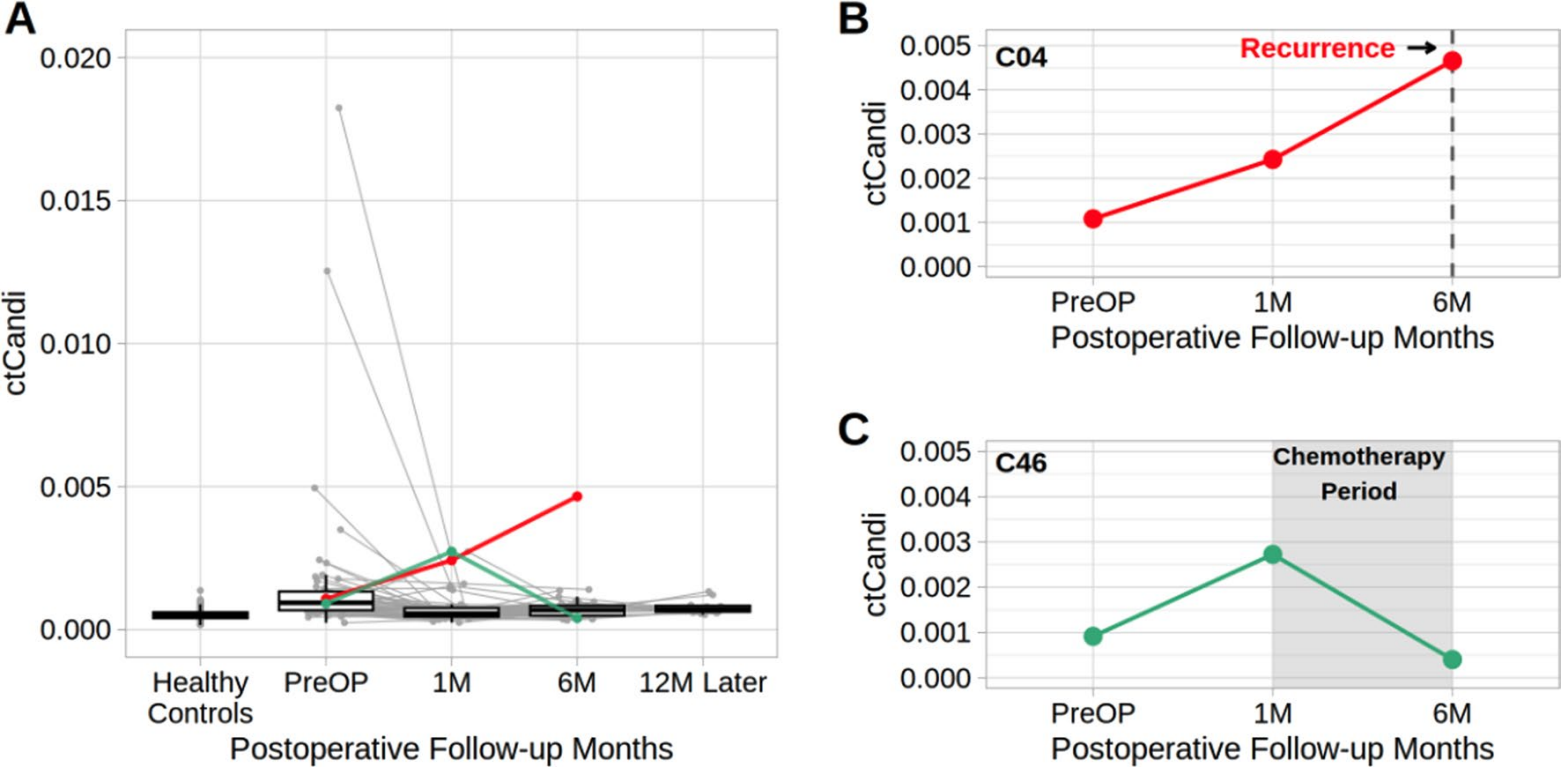
Monitoring of prognosis after surgical resection for colon cancer patients based on ctCandi. **A** Changes in ctCandi values from before operation to 12 months after operation. Gray lines indicate ctCandi of individual patients excluding C04 and C46. Red line indicates ctCandi values of C04 patients. Green line indicates ctCandi values of C46 patients. **B**, **C** ctCandi of individual patients before and one month and six months after the operation. Dashed line and shaded region indicate recurrence and postoperative chemotherapy, respectively

To assess the clinical potential of our approach in postoperative monitoring, we applied CaSH-based ctCandi to longitudinally collected cfDNA from colon cancer patients. We found a reduction of ctCandi values in 81.6% (*N* = 40) of all patients at one month post-operation compared to preoperative levels (Fig. 4A). Additionally, patients without recurrence between 1 and 12 months after the operation also decreased ctCandi values compared to preoperative levels (Fig. 4A). In contrast, there were two cases where ctCandi unexpectedly increased one month after the operation. For instance, in the case of patient C04, distant recurrence in the liver occurred within six months after the operation, and this patient’s ctCandi value continuously increased from pre-operation to six months after the operation (Fig. 4B). The patient died after the recurrence. Another notable case is patient C46 who showed an increase in ctCandi at one month after the operation. However, after completing six months of postoperative chemotherapy starting from the first month after the operation, ctCandi values decreased compared to the preoperative levels. Subsequently, this patient has not experienced recurrence in the six months following the completion of chemotherapy (Fig. 4C).

In addition to patient C04, there were five more cases of recurrence post-operation (Fig. S9). Patient C34 showed a sustained increase in ctCandi from one to ten months post-operation, with distant recurrence to the lung occurring in the 14th month after the operation (Fig. S9A). The ctCandi levels of other metastatic recurrence—patients C03 (liver metastasis), C11 (lung metastasis), C16 (liver metastasis) and C22 (liver and peritoneum metastasis) increased from the first to the sixth month after operation. Unfortunately, we were not able to measure whether there was an elevation in ctCandi at the time of proximity to the recurrence because of the absence of the samples (Fig. S9B–E).

Interestingly, we found that a region (chr6:391,824–393,789, 1966 bp) on exon2 of *IRF4* (interferon regulatory factor 4) contributed a major role in the reduction of ctCandi when we identified the CaSH regions associated with the decrease in ctCandi across 22 individual patients before and one month after the operation (Fig. S10A; Pairwise Wilcoxon rank-sum test, *P* = 1.2 × 10^–4^). The methylation and expression level of *IRF4* have been reported to be associated with the development of colon cancer [28]. Hypermethylated markers on *IRF4* have been reported as useful for detecting colorectal cancer patients using cfDNA [29].

Furthermore, a region (chr13:93,227,291–93,228,759, 1469 bp) on exon 1 of *GPC6* (Glypican 6) exhibited a significant decrease in ctCandi across 51% (*N* = 25) of the overall patients (Fig. S10B; Pairwise Wilcoxon rank-sum test, *P* = 1.3 × 10^–2^). The hypermethylation and down-regulated mRNA expression of the *GPC6* have consistently been observed in colon cancer, as reported in a previous study [30]. We also confirmed that *IRF4* and *GPC6* show a significantly negative correlation between the average *β* values and transcript per million (TPM) values from TCGA dataset (Spearman’s correlation, *IRF4*: *ρ* = − 0.38, *P* = 2.9 × 10^–10^; *GPC6*: *ρ* = −0.31, *P* = 3.3 × 10^–7^; Fig. S11).

## Discussion

Our study has several critical limitations. First, the defined CaSH regions have not yet been validated for cancers other than colon cancer. As a result, the generalizability of this CaSH concept is uncertain. Despite this, the regions show promising clinical utility in detecting and monitoring colon cancer. Additionally, while we have not conducted a comparative analysis to identify methylation profiles specific to all cancer types, the defined regions might encompass pan-cancer methylation profiles. Therefore, future studies should focus on selecting more definitive colon cancer-specific methylated regions through comparative analysis of methylation profiles from various cancers. Second, our classification model, based on a small sample of 49 colon cancer patients, requires further validation with an independent cohort. Third, our study's credibility is limited by a short six-month clinical follow-up period. To fully assess ctCandi's reliability in clinical decision-making, especially in predicting patient recurrence, we need more extensive, long-term follow-up data. Lastly, the lack of blood samples at recurrence times limits our ability to accurately gauge ctCandi's effectiveness in detecting recurrence. To validate ctCandi's feasibility in monitoring cancer patients, additional blood samples should be collected both at the time of recurrence and in periods closely preceding it.

One last discussion point we would like to mention is cancer detection using hypomethylated regions. We failed to acquire high performance using hypomethylated regions when we tested a ctDNA quantification method based on the CaSH region in diagnosing patients. We think this lack of predictive information stems from the fact that in most normal human cells, promoter regions are predominantly unmethylated [31, 32], creating significant background noise that interferes with our method's ability to provide cancer-specific hypomethylation information. We observed that while CaSH regions with 10 or more hypermethylated CpGs are common enough to generate cancer-related signals, there are significantly fewer instances of 10 or more tandemly occurring hypomethylated regions that are specific to colon cancer.

## Conclusion

We introduced a method for detecting and monitoring colon cancer using cell-free DNA methylation profiling, focusing on 901 cancer-specific hypermethylated regions. This method effectively identifies colon cancer-specific methylated ctDNA, eliminating false signals from blood-derived cfDNA. It offers sensitive ctDNA quantification in blood, enhancing the identification of colon cancer patients and tracking their response to treatments. This approach may provide crucial improvement in liquid biopsy-based patient care, reducing the need for invasive tests and aiding in early recurrence prediction.

## Materials and methods

### Patient recruitment and clinical characteristics

We collected tissue and blood plasma samples from 49 patients diagnosed with stage II or III colon cancer at the Dongnam Institute of Radiological and Medical Sciences (DIRAMS), Busan, Republic of Korea. This study received approval from the Institutional Review Board (IRB) of DIRAMS (IRB No.: D-2003-011-002). In total, 94 tissue samples were collected, including 47 tumor tissue samples from colon cancer patients and 47 matching normal tissues from adjacent regions to the tumor. However, we could not acquire tissue samples from two patients due to their small size. Plasma samples corresponding to the 49 patients were collected at four time points per patient: before the operation (*N* = 49), one month after the operation (*N* = 49), six months after the operation (*N* = 46) and more than 12 months after the operation (*N* = 16; one sample was exceptionally collected at 10 months post-operation). Samples were initially collected six months post-operation, followed by regular six-month interval collections, with varying success rates depending on each patient (Fig. S3). The median follow-up period for recurrent and nonrecurrent patients was 20 months, and the median time to recurrence for patients with recurrence was 13 months. Among the total colon cancer patients, 36.7% (*N* = 18) were diagnosed with stage II and 63.3% (*N* = 31) with stage III. Additionally, 12.2% (*N* = 6) of patients experienced distant recurrence after the operation (Table 1). A healthy control group consisted of 260 plasma cfDNA samples selected from the Korean Genome Project (KGP), approved by the Institutional Review Board (IRB) at UNIST in Ulsan, South Korea (IRB No.: UNISTIRB-21-66-A). The participants in this group had no history of cancer, and the female participants were not pregnant.

### Sample processing

To extract DNA from the tissue samples, we pulverized the tissue using a mortar and pestle in liquid nitrogen. The resulting powder was then homogenized in a cell lysis solution consisting of 2% CTAB, 1.4 M NaCl, 100 mM Tris–Cl (pH 8.0), 20 mM EDTA and β-mercaptoethanol (added immediately before use at a ratio of 100 μl per 10 ml). After thorough mixing, proteinase K was added, and the mixture was incubated at 65 °C for 1.5 h. Subsequently, an equal volume of phenol–chloroform-isoamyl alcohol (25:24:1, PCI) was added to the lysate, followed by centrifugation at 12,000 rpm for 10 min at room temperature. We then isolated the top aqueous phase and incubated it at 37 °C for 1 h after adding RNase A at a concentration of 100 μg/ml. Following this, an equal volume of chloroform-isoamyl alcohol (24:1) was added, and the mixture was centrifuged under the same conditions. The supernatant was collected, to which 1/12 volume of 5 M NaCl and twice the volume of 100% ethanol were added. After 30 min of incubation at − 20 °C, the DNA pellet was collected by centrifugation, washed with 70% ethanol and finally dissolved in 100 μl of ion-exchanged ultrapure water. In the Korean Genome Project (KGP), plasma samples were obtained by initially separating plasma from whole blood using Cell-Free DNA BCT tubes (Streck). This separation was achieved by centrifuging at 1500 g for 10 min at room temperature, followed by a subsequent centrifugation at 3000 g for 10 min at 4 °C to remove any remaining cells. Cell-free DNA was extracted from 3 to 5 ml of plasma using the QIAamp Circulating Nucleic Acid Kit (QIAGEN, 55,114) according to the manufacturer's instructions. The concentration of cfDNA was measured using the Qubit dsDNA HS Assay Kit (Thermo Fisher Scientific), and its quality was evaluated using the 4150 TapeStation system (Agilent Technologies). Only samples with cfDNA purity of 80% or more and a total amount of 5 ng or more were used for this study.

### Sequencing library preparation

Enzymatic conversion for DNA library preparation was carried out following the protocol provided by the NEBNext® Enzymatic Methyl-seq Kit (NEB). In summary, the process began with the ligation of cfDNA at a concentration ranging from 5 to 10 ng with amplification adaptors that featured methylated cytosines. This was followed by DNA fragmentation end repair and A-tailing. Subsequently, in the initial step of enzymatic conversion, the adaptor-ligated DNA was subjected to oxidation facilitated by TET2 and an oxidation enhancer. This step aimed to protect 5-methylcytosine and 5-hydroxymethylcytosine from potential deamination in subsequent stages. In the second phase of enzymatic conversion, APOBEC was utilized to convert cytosine into uracil, while ensuring that the oxidized forms of 5-methylcytosine and 5-hydroxymethylcytosine remained stable. Following this, the enzymatically modified DNA underwent an amplification process using sequencing index primers, involving eight cycles of PCR amplification as per the provided guidelines. At each stage of the process, DNA purification was executed with the utilization of NEBNext sample purification beads, following the manufacturer's recommended protocol. All constructed libraries were quantified using the Qubit dsDNA HS Assay Kit (Thermo Fisher Scientific) and the D1000HS tape with the 4150 TapeStation system (Agilent Technologies). Paired-end 150 bp reads from these libraries were sequenced on the Illumina Novaseq 6000 platform.

### Sequencing data processing

We performed enzymatic methylation sequencing (EM-seq) [33] on the collected samples with 53.72 Gbp (Giga base pair) on average. FASTQ files were generated from the EM-seq libraries using Illumina NovaSeq 6000 system. Illumina adapter sequences and poly-*g* tails were trimmed by fastp (ver. 0.20.1), and low-quality reads, which have a lower average Phred quality score than 20 or are shorter than 20 bp or N-bases more than 2, were filtered by fastp (ver. 0.20.1) [34]. The preprocessed reads were aligned to the bisulfite-converted hg38 reference genome sequence using Bismark (ver. 0.22.3) [35]. In this alignment step, unpaired or not uniquely mapped reads were removed by Bismark (ver. 0.22.3). Finally, duplicate reads were removed by MarkDuplicates in Picards (ver. 2.25.0). These preprocessed BAM files were used for the following analysis.

### Definition of genome-wide colon cancer-specific hypermethylated (CaSH) regions

The discovery cohort for identifying genome-wide colon cancer-specific hypermethylated (CaSH) regions consisted of 41 cancer and 47 normal colon tissues. The methylation ratios (*β* values) of genome-wide CpG sites were obtained from BAM files using BismarkExtractor (ver. 0.22.3). These *β* values were merged into a single value for each CpG site using bedtools (ver. 2.29.1), based on the Cytosine (C) positions in the hg38 human reference genome. Colon cancer-specific hypermethylated CpGs were defined as those with a difference in *β* value (*β*_tumor tissue_–*β*_normal tissue_ > 0.3) and a false discovery rate (FDR) < 0.05 between cancer and normal tissues. The most critical step involved excluding methylated CpGs in the healthy controls, which had *β* value thresholds higher than 0.05 in 190 healthy plasma samples. This step enhanced the sensitivity and specificity of our CpG marker selection by comparing cancer-specific methylation patterns with those of healthy controls. Additionally, we excluded CpG sites with *β* values unavailable in more than half of the samples. Considering the maximum read length of 150 bp, we merged adjacent hypermethylated CpG sites within a 75 bp range up/downstream, forming a CaSH fragment. Fragments containing fewer than ten CpGs were excluded to maintain the sensitivity of ctDNA quantification. Finally, the region encompassing the first and last CpG in the merged CaSH fragment was defined as a CaSH region.

### Identification of differentially hypomethylated CpGs and regions in colon cancer

The differentially hypomethylated CpGs were defined as CpGs with a difference in *β* value (*β*_normal tissue_–*β*_tumor tissue_) > 0.3 and FDR < 0.05 between cancer and normal tissue. Following that, to enhance the sensitivity and specificity of defining cancer-specific hypomethylated CpGs, we excluded methylated CpGs based on *β* value thresholds of lower than 0.95 in 190 healthy plasma samples. The process of defined regions was the same as the previous section.

### Calculate ctDNA candidate count index (ctCandi)

CaSH-based ctCandi counts reads with a read methylation density (RMD) higher than 0.6, while being completely contained within the CaSH region. In hypomethylated regions, reads with a lower read methylation density than 0.3 were classified as candidate ctDNA reads. The number of candidate ctDNA reads for each CpG site was normalized using CPM (counts per million mapped reads). The ctCandi was calculated as the average of the normalized candidate ctDNA count of all regions.

### Validation of CaSH regions using the Cancer Genome Atlas (TCGA) and gene expression omnibus (GEO)

CaSH regions were validated using Infinium Methylation 450 K array data obtained from the Gene Expression Omnibus (GEO) and The Cancer Genome Atlas (TCGA), comprising 263 colon tumor tissue samples and 35 colon normal tissue samples from TCGA, along with 656 healthy blood samples from GSE40279. CpG sites with *β* values unavailable in more than half of the samples were excluded. Subsequently, CpG sites on the Methylation 450 K array were classified into those overlapping with the CaSH regions and those that did not overlap. Average *β* values of CpG sites were calculated for each group. Rank-sum test was conducted to compare between the groups.

### Correlation between gene expression and methylation

We examined the association between methylation levels and transcription levels of genes *IRF4* and *GPC6* in the CaSH region in colon cancer patients. We utilized 258 colon cancer tissue samples from TCGA, each with available Infinium Methylation 450 K array and RNA-seq data. Then, Spearman’s correlation analysis was performed to calculate the correlation between methylation and expression levels using the overlapping CpG sites of these genes.

### in silico validation

We performed an in silico test to evaluate the quantitative correlation of ctCandi on colon cancer. To generate raw in silico data, we use ten healthy plasma data and six colon tissue samples considering age and sex. During all this process, splitting and merging reads from BAM files were carried out using GATK (ver. 4.19.0) [36]. We generated six simulated healthy plasma data containing approximately 10 × reads from the entire healthy plasma reads. Using the data, the mixture ratios ranged from 0.1% to 5% and six sets of simulated data were created for each ratio. We calculated ctCandi using these in silico data. Through Spearman’s correlation analysis, we identified a significant positive correlation between the proportion of cancer tissue and ctCandi using the in silico data.

### Gene annotation and enrichment analysis

A total of 797 genes were identified through annotation using the R package annotateR (ver. 1.20.0) [37], based on 901 CaSH regions. Subsequently, the enrichment analysis was conducted for these regions. The enrichment analysis utilized the shinyGO (ver. 0.77) [38]. For gene ontology analysis, the focus was on the ‘Molecular Function’ category, using data from Ensembl version 92. In the gene set enrichment analysis, we used the result of MethCancerDB in the other category [39].

### Model construction

We constructed the logistic regression model based on the methylation signature of cfDNA to distinguish colon cancer from healthy control. The normalized ctDNA candidate counts about the regions were used as input features. The models were performed three fold cross-validation with 60 healthy controls and 49 colon cancer patients. The models were conducted without the penalty, and all remaining options were default settings.

### Bioinformatic and statistical software

Scripts for data analysis were written in Python3. The python package Pysam (ver. 0.21.0) was used to compute methylation from the reads in BAM files. The python package Pandas (ver. 1.5.3) was used for manipulation of tabular data. The python package Scipy (ver. 1.11.1) was used for statistical analysis, including the calculation of Spearman’s correlation coefficients and a Wilcoxon rank-sum test. The python package Sklearn (ver. 1.2.2) was used to construct the machine learning models. For generating graphs and figures, R package ggplot2 (ver. 3.4.4) and grid (ver. 4.2.0) packages were used (R version 4.2.0).

### Supplementary Information

Additional file1 (DOCX 2349 KB)

## Data Availability

The datasets used in the current study are available from the corresponding authors upon request.

## List of abbreviations

AUC: Area under curve
CaSH region: Cancer-specific hypermethylated region
CEA: Carcinoembryonic antigen
cfDNA: Cell-free DNA
ctCandi: CtDNA candidate count index
ctDNA: Circulating tumor DNA
GO: Gene ontology
KGP: Korean Genome Project
MSI: Microsatellite instability
MSS: Microsatellite stable
RMD: Read methylation density
