# Supplementary material for: Quantification method of ctDNA using cell-free DNA methylation profile for noninvasive screening and monitoring of colon cancer
Source: Clin Epigenetics. 2024 Jul 19;16:95. doi: 10.1186/s13148-024-01708-9 (PMC11264732; doi:10.1186/s13148-024-01708-9)
Supplement: Supplementary file 1 — Additional file1 (DOCX 2349 KB) [file 13148_2024_1708_MOESM1_ESM.docx]

**Quantification method of ctDNA using cell-free DNA methylation profile for non-invasive screening and monitoring of colon cancer**

**Supplementary Information**

**
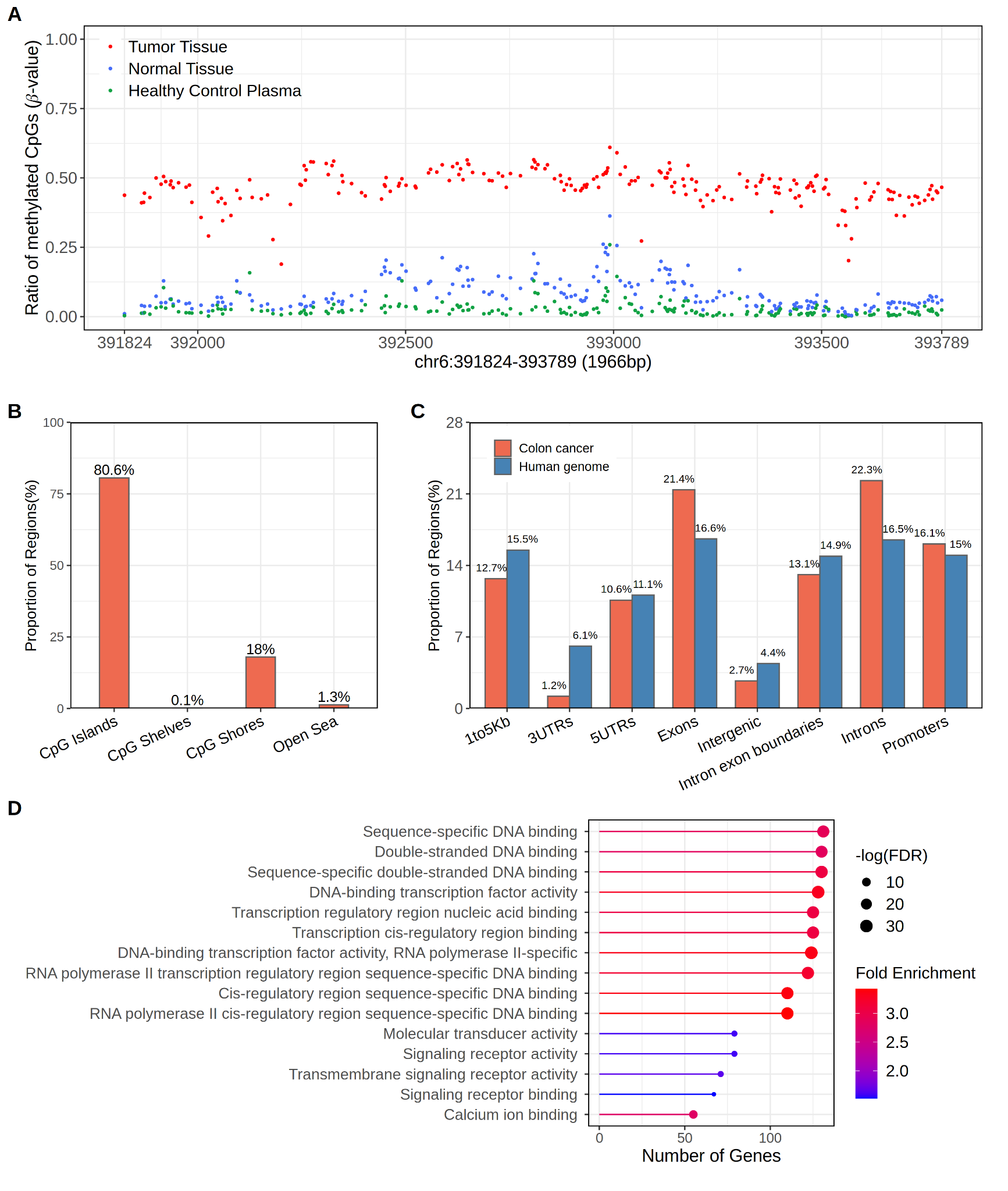
**

**Figure S1.** Identification of colon cancer-specific hypermethylation regions. **A)** The longest region among the 901 colon CaSH region. **B)** and **C)** Distribution of genomic location of the selected regions. **D)** GO enrichment analysis result of molecular function.

**
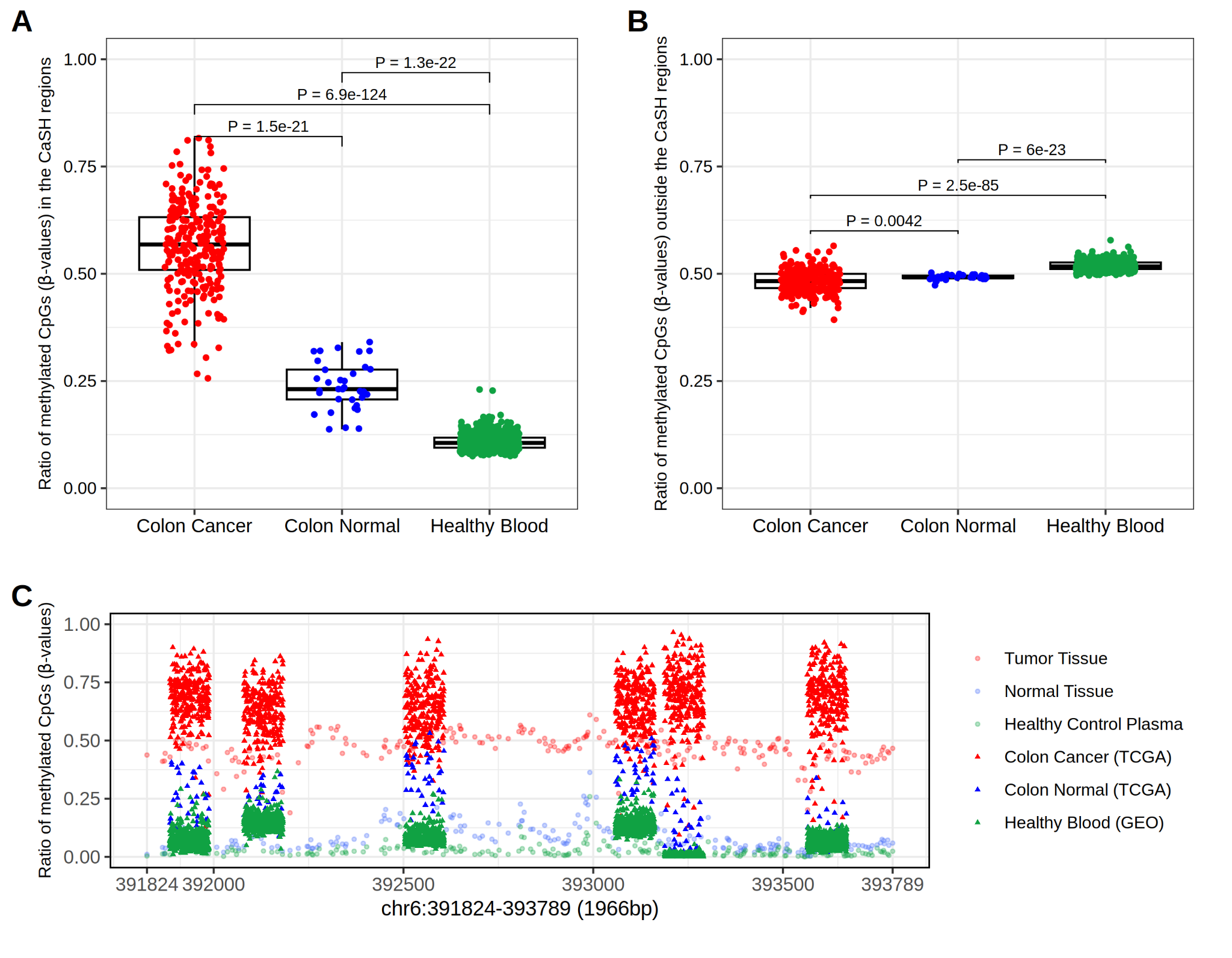
**

**Figure S2.** Validation of CaSH regions. A) Ratio of methylated CpG sites among 1,676 CpG sites in 681 CaSH regions between 263 colon cancer tissues, 35 colon normal tissues, and 656 healthy blood samples. B) Ratio of methylated CpG sites among 439,311 CpG sites outside CaSH regions between 263 colon cancer tissues, 35 colon normal tissues, and 656 healthy blood samples. C) Ratio of methylated CpG sites among 6 CpG sites in the longest CaSH region between 263 colon cancer tissues, 35 colon normal tissues, and 656 healthy blood samples. *P-*values were calculated by the Wilcoxon rank sum test.


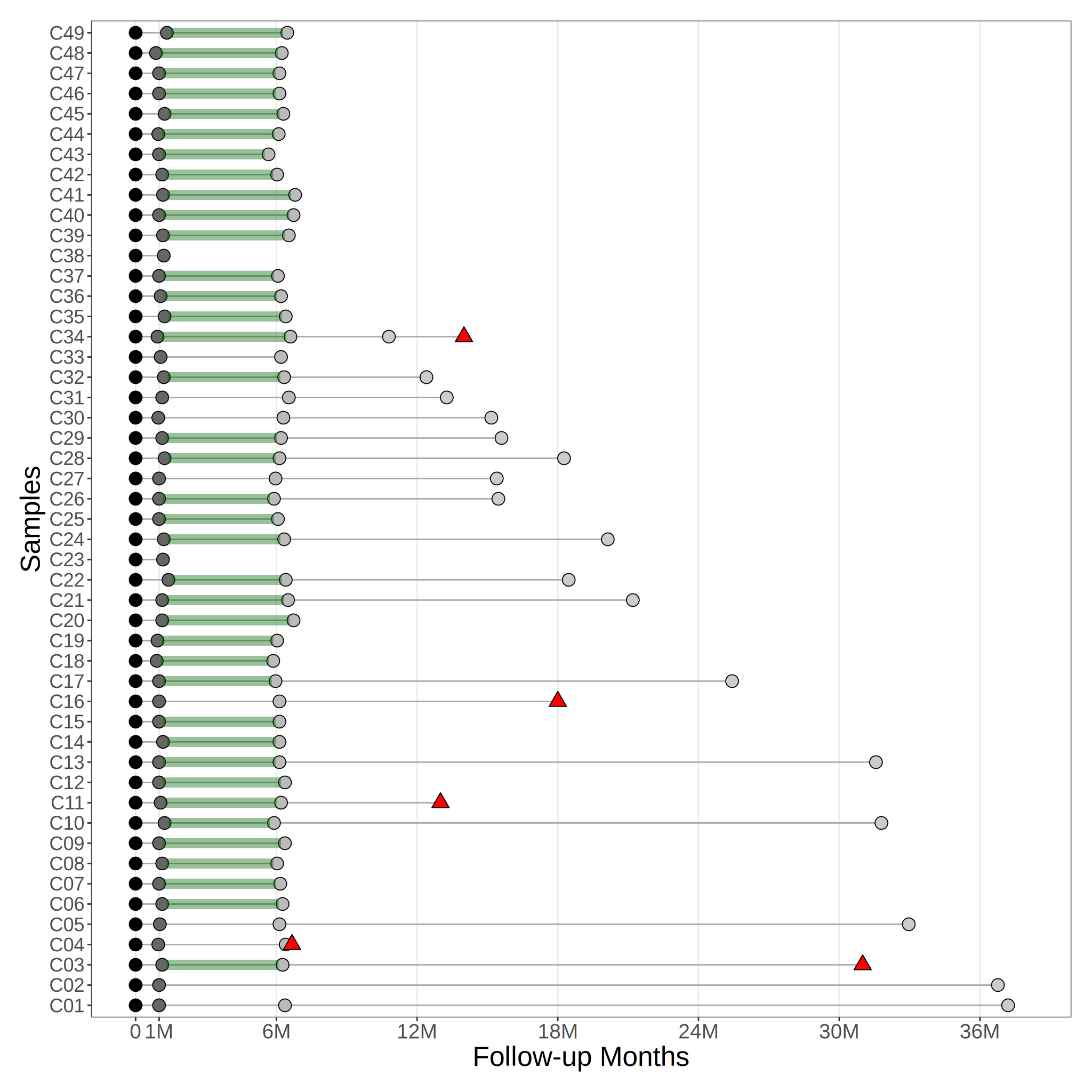


**Figure S3.** Clinical follow-up duration of 49 colon cancer patients. The green thick line and red triangle represent the chemotherapy period and recurrence, respectively.

**
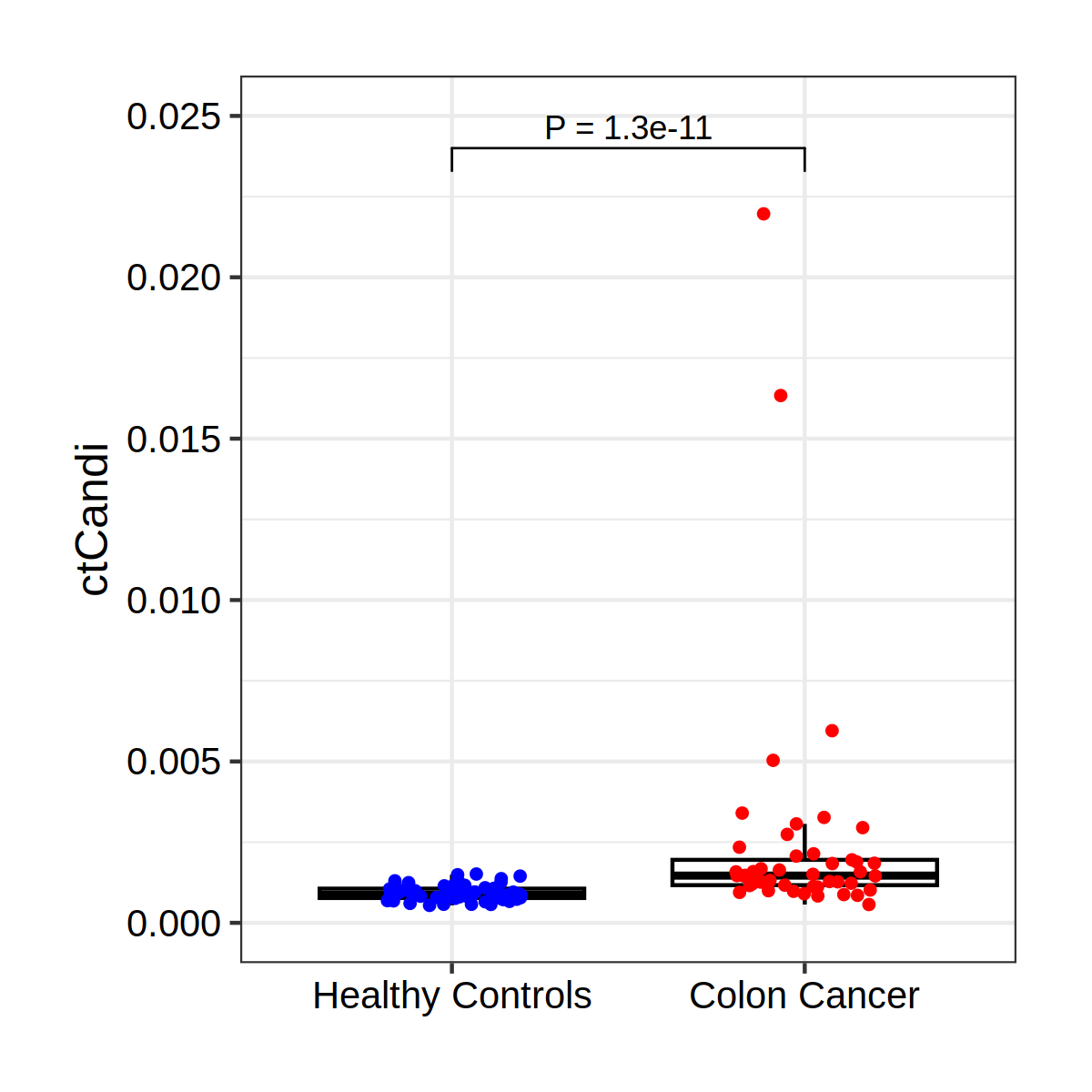
**

**Figure S4.** ctCandi calculated from 29,557 hyper methylated CpG sites between 60 healthy controls and 49 colon cancer patients. The *P-*value was calculated by the Wilcoxon rank sum test.

**
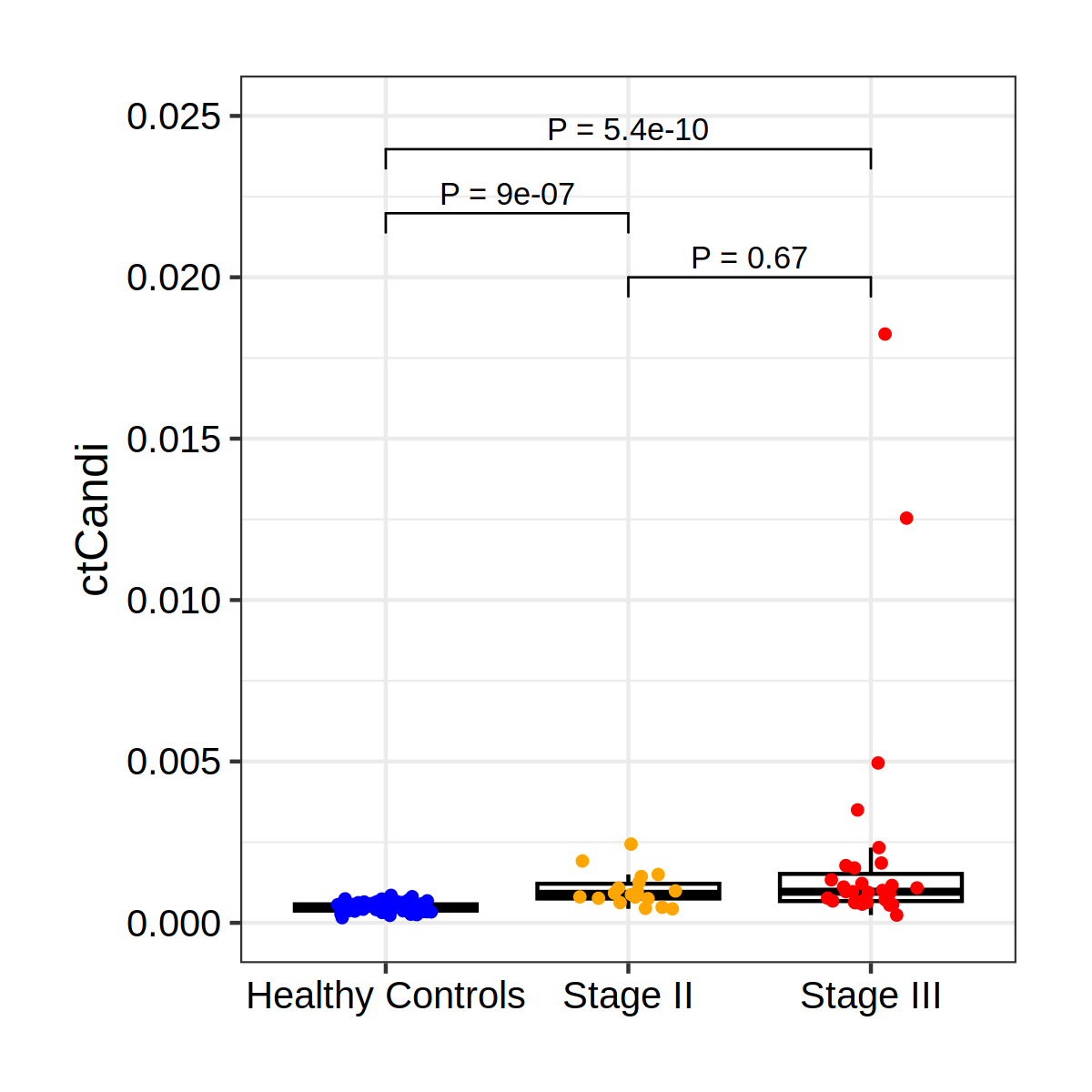
**

**Figure S5.** Comparison of CaSH-based ctCandi between 60 healthy controls and 49 colon cancer patients according to cancer stages. *P-*values were calculated by the Wilcoxon rank sum test.


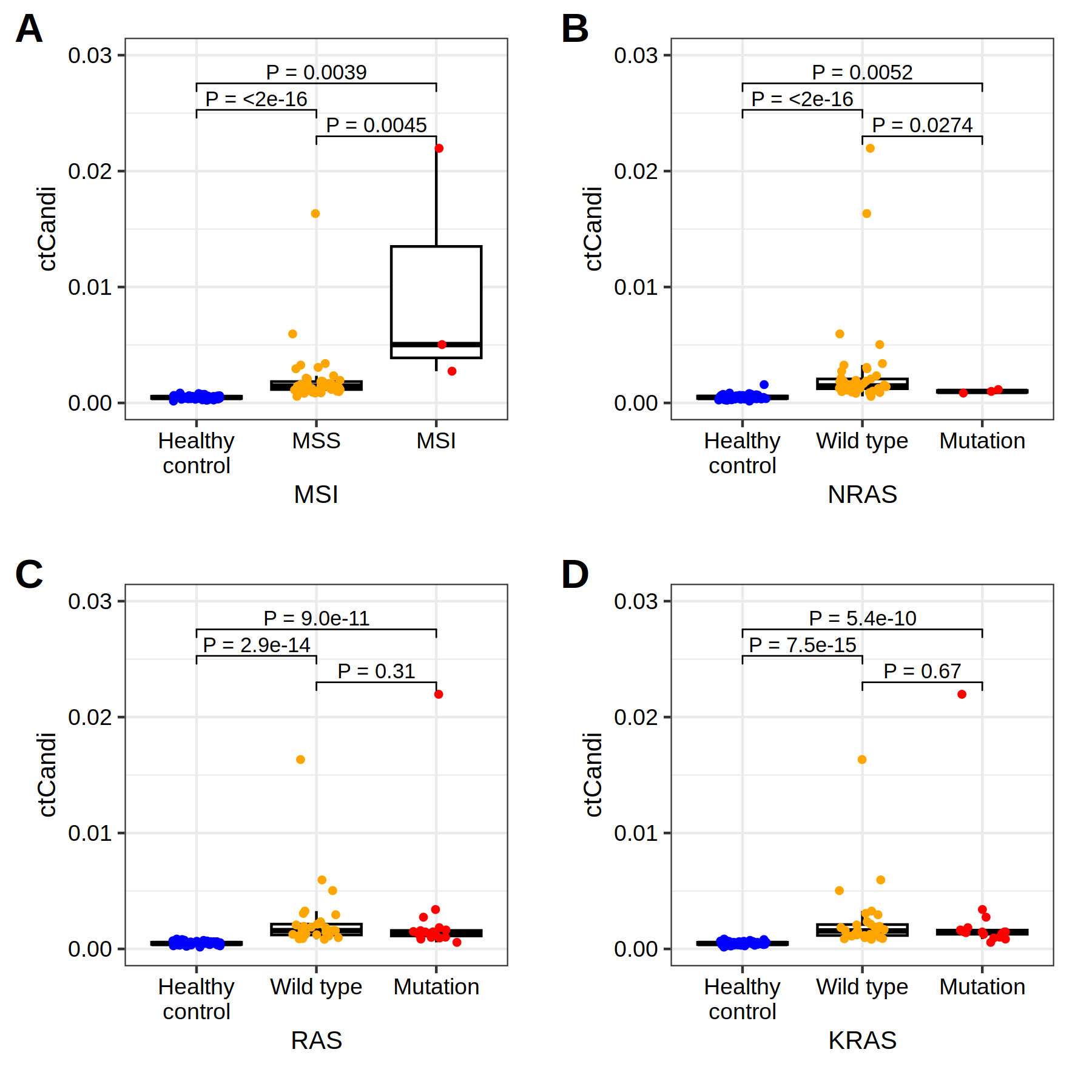


**Figure S6.** Comparison of CaSH-based ctCandi between MSI, RAS, KRAS and NRAS in 49 colon cancer patients. *P-*values were calculated by the Wilcoxon rank sum test.

**
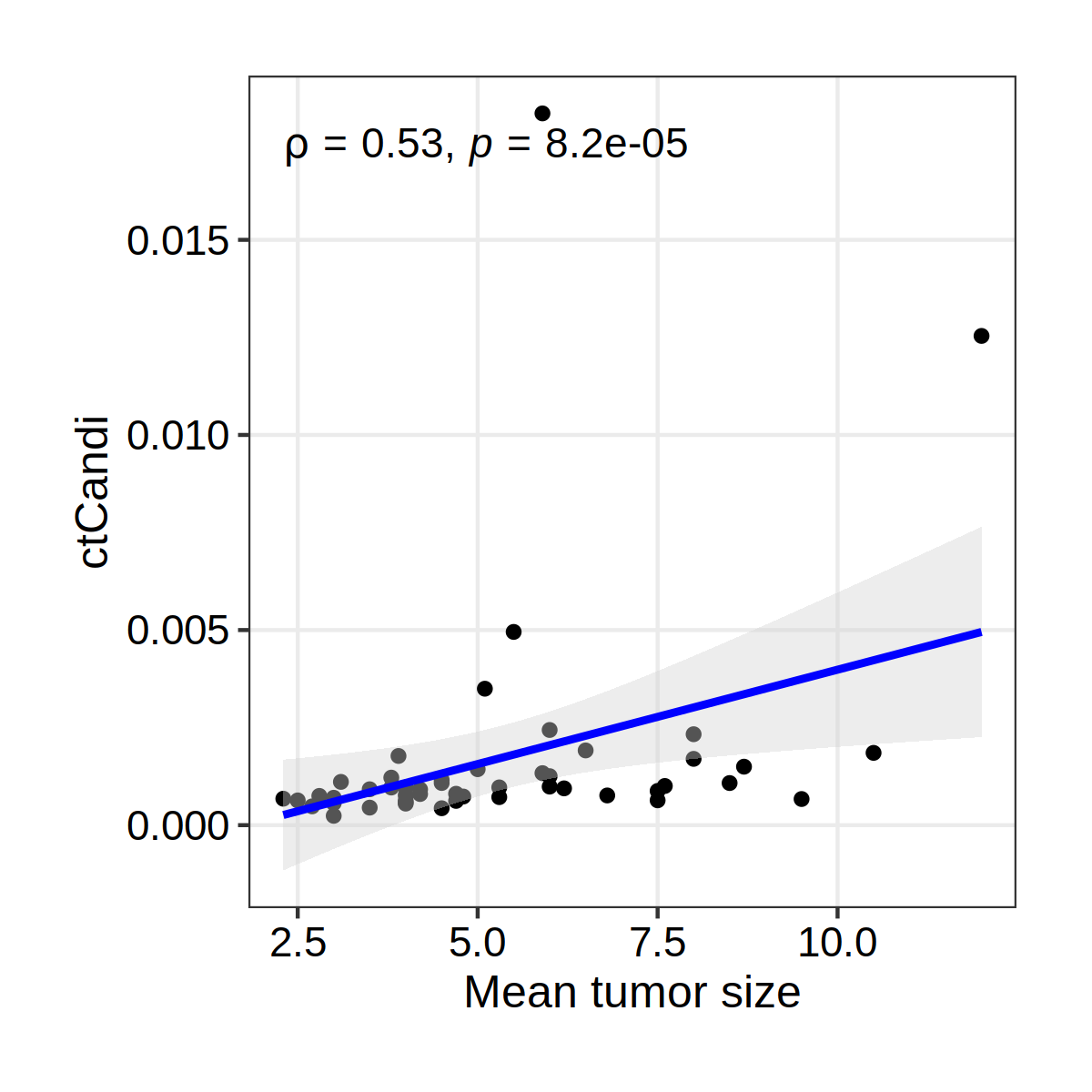
**

**Figure S7.** Spearman correlation between ctCandi and tumor size in colon cancer patients. The blue line represents the regression line and the shaded region in light gray indicates the confidence region.


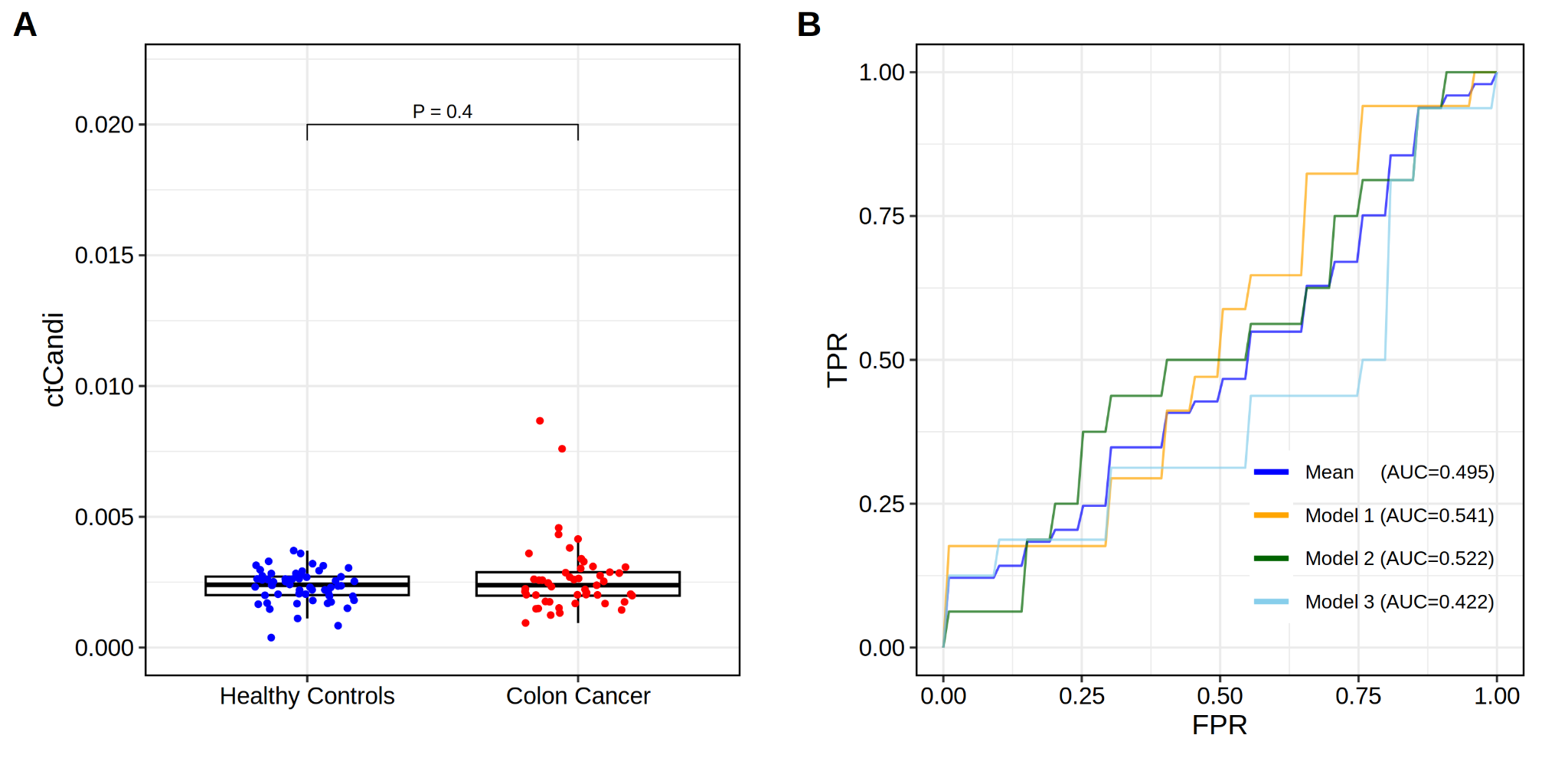
**Figure S8.** Performance of ctCandi and classification machine learning models using 48 differentially hypomethylated regions in colon cancer. **A)** CtCandi of 49 colon cancer patients and 60 healthy controls **B)** Receiver operating characteristic (ROC) curves of ctCandi for distinguishing the colon cancer patients and the healthy controls. TPR indicates a true positive ratio, and FPR indicates a false positive ratio.

**
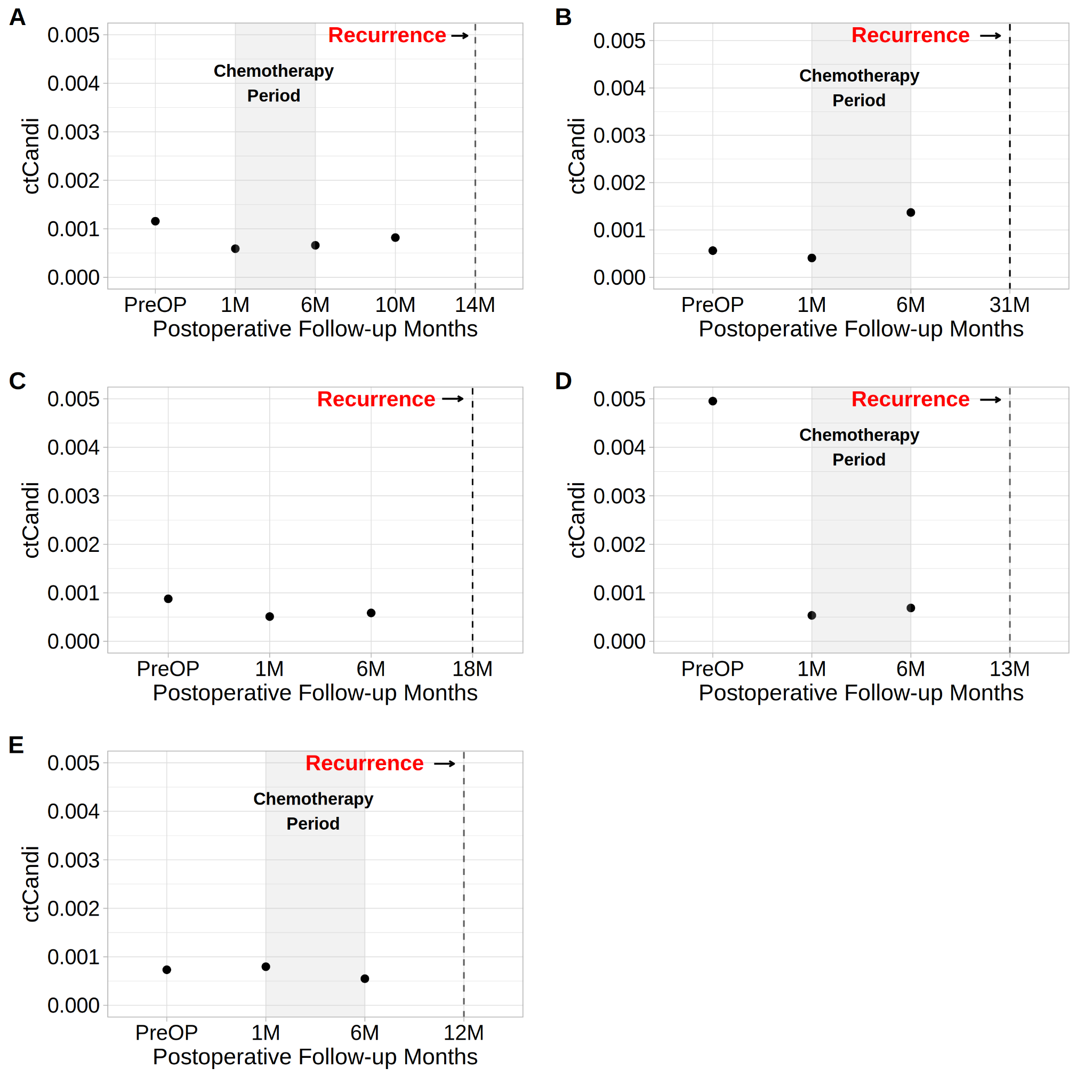
**

**Figure S9.** Postoperative ctCandi measurements in five recurrence cases. **A-E)** Dashed line indicates time of recurrence. The shaded region in light gray indicates the period of postoperative chemotherapy. **A)** C34 **B)** C03 **C)** C16 **D)** C11 **E)** C22

**
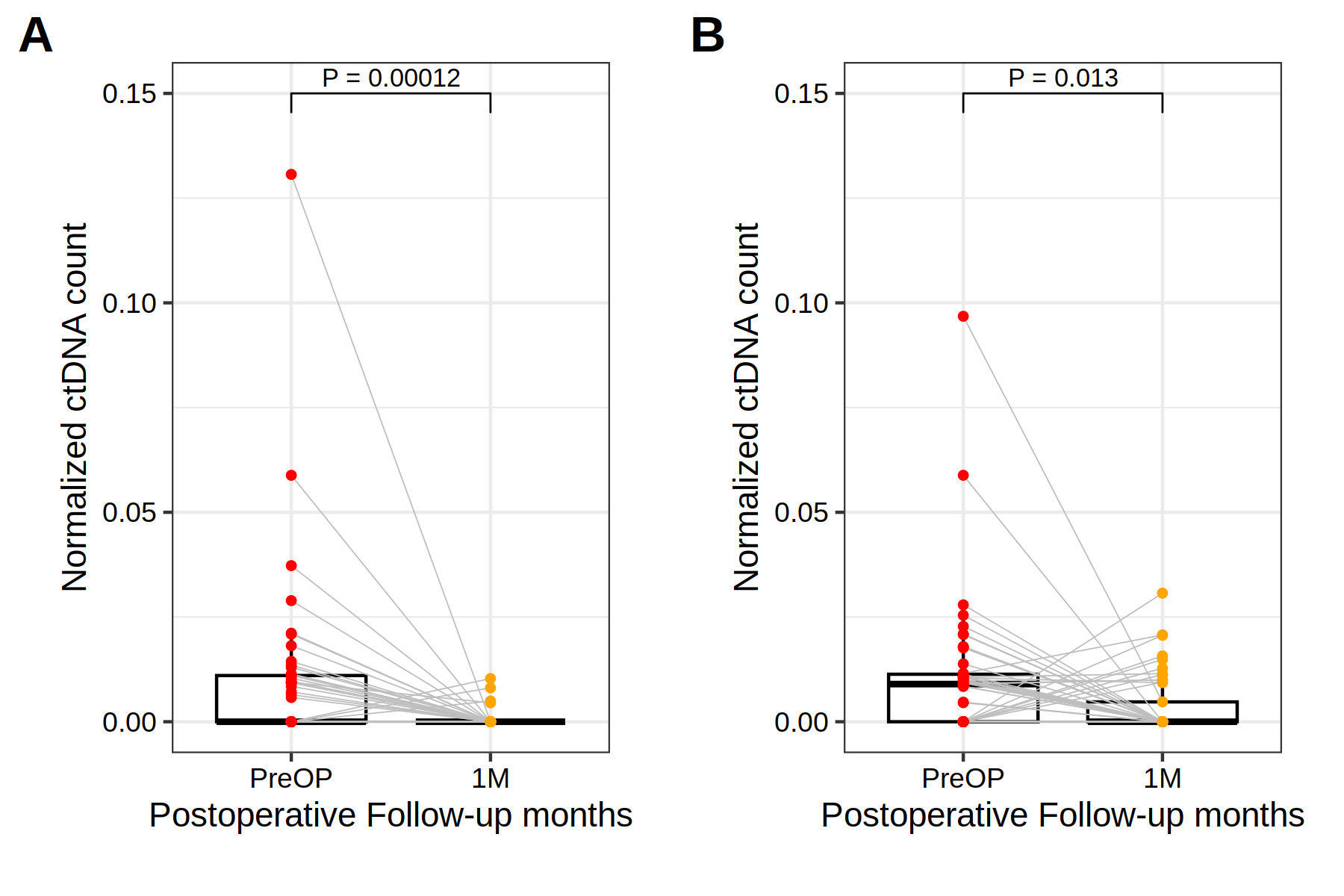
**

**Figure S10.** Reduction in ctDNA associated with colon cancer-specific hypermethylated regions located in two genes. **A)** ctDNA count changes in *IRF4* (chr6:391824-393789, 1966 bp). **B)** ctDNA count changes in *GPC6* (chr13:93227291-93228759, 1469 bp)


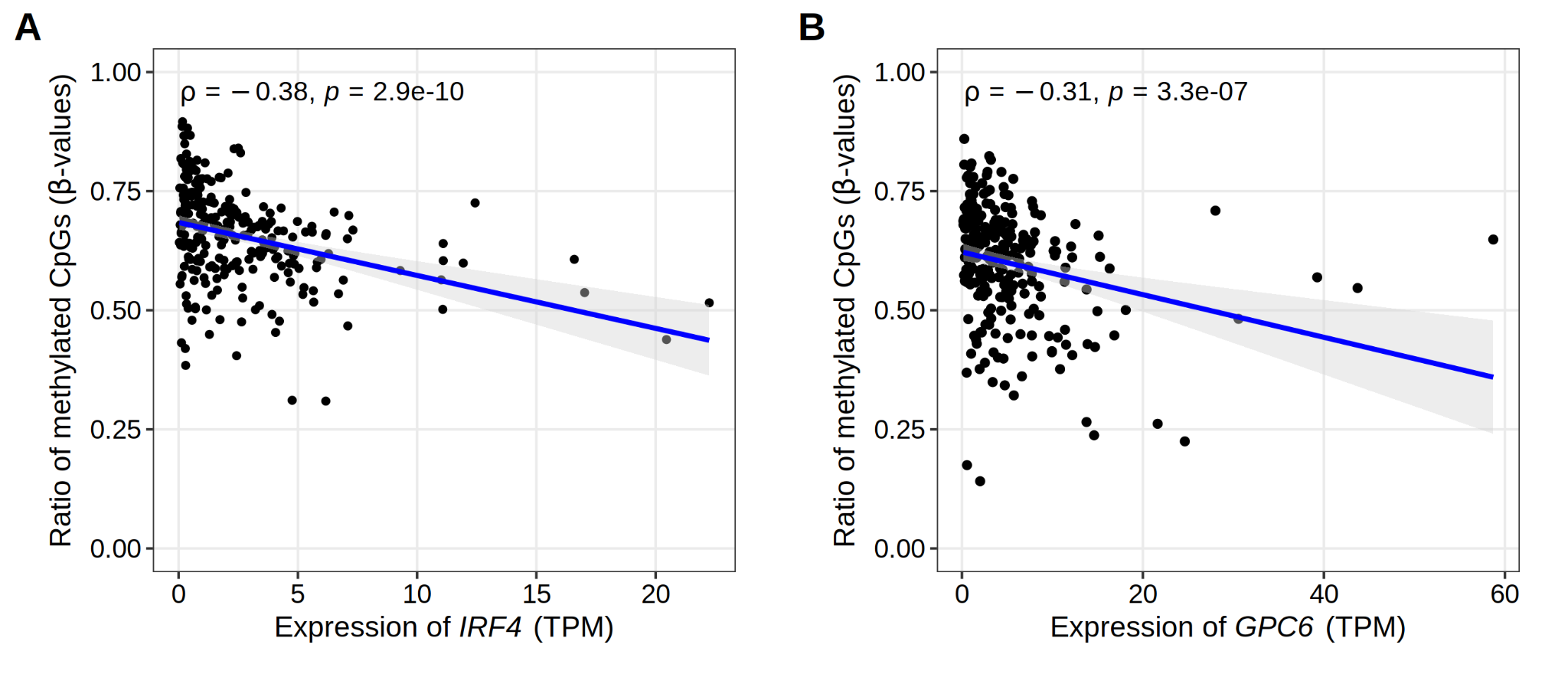


**Figure S11.** Correlation between methylation and expression level in colon cancer. A) *IRF4*. B) *GPC6.* The blue line represents the regression line, and the shaded region in light gray indicates the confidence region.
